# Phytochemical Constituents and the Evaluation Biological Effect of *Cinnamomum yabunikkei* H.Ohba Leaf

**DOI:** 10.3390/molecules24010081

**Published:** 2018-12-27

**Authors:** Seung-Yub Song, Seung-Hui Song, Min-Suk Bae, Seung-Sik Cho

**Affiliations:** 1 Department of Pharmacy, College of Pharmacy, Mokpo National University, Muan, Jeonnam 58554, Korea; tgb1007@naver.com (S.-Y.S.); tmdgml7898@naver.com (S.-H.S.); 2 Department of Environmental Engineering, Mokpo National University, Muan, Jeonnam 58554, Korea; minsbae@hotmail.com

**Keywords:** *Cinnamomum yabunikkei* leaf, antioxidant, xanthine oxidase, elastase

## Abstract

*Cinnamomum yabunikkei* H.Ohba leaf is known as a traditional medicinal material in Korea. However, no scientific identification of the components or efficacy of *C.yabunikkei* H.Ohba leaf has been reported. In the present study, we prepared various solvent extracts of *C.yabunikkei* H.Ohba leaf to understand its basic properties and evaluated the antioxidant, xanthine oxidase inhibitory, and elastase inhibitory activities of hexane, ethyl acetate, acetone, methanol, ethanol, and water extracts for the first time. The antioxidant properties were evaluated based on 1,1-diphenyl-2-picrylhydrazyl (DPPH) free radical scavenging activity, reducing power, and total phenolic contents. The hot water extract showed the highest DPPH radical scavenging activity and total phenolic contents, and the reducing power was the highest in the water extract. The hexane extract showed an excellent elastase inhibitory effect compared to control (phosphoramidone) and the highest xanthine oxidase inhibitory activity. These results present basic information for the possible uses of the hot water and hexane extracts from *C. yabunikkei* leaf for the treatment of diseases caused by oxidative imbalance. In the present study, individual extracts exhibited different effects. Therefore, it is hypothesized that the applicability of *C. yabunikkei* will depend on the extraction method and nature of the extract. The hot water and hexane extracts could be used as antioxidants, and as anti-gout and anti-wrinkle materials respectively. Several biologically active substances present in hexane extract of *C. yabunikkei* have been analyzed by GCMS and demonstrated to possess antioxidant and xanthine oxidase inhibitory activity. To the best of our knowledge, this is the first study that reports the chemical profiling and biological effects of various *C. yabunikkei* leaf extracts, suggesting their potential use in food therapy, cosmetics or alternative medicine.

## 1. Introduction

*Cinnamomum yabunikkei* H.Ohba is a species of camphor tree native to the southern coast and Jeju Island in South Korea and can be seen in southern China and Taiwan. Leaves have been used as a bath material or tea and berries as a source of oil. In addition, the bark has traditionally been used for the treatment of various diseases related to pain and blood circulation.

Due to the acceleration in global warming, the southern part of South Korea has experienced the cultivation of *C.yabunikkei* H.Ohba, hence studies dealing with the possibilities of using *C.yabunikkei* H.Ohba for various purposes are necessitated.

Until now, the components of the leaves of *C.yabunikkei* have not been identified and biological activity studies have not been conducted. To the best of our knowledge, this is the first report to understand the composition and biological activity of the *C.yabunikkei* leaf.

In the present study, various extracts of the *C.yabunikkei* leaf using hexane, ethyl acetate, acetone, ethanol, methanol, and hot water were prepared to determine the optimal extraction solvent with respect to biological activity and phytochemical profiles. Gas chromatography-mass spectrometry (GC-MS) was used for the chemical profiling of hexane extracts. Subsequently, antioxidant, xanthine oxidase inhibitory, and elastase inhibitory activity of the leaf extracts were examined. The antioxidant activity was confirmed by measuring 1,1-diphenyl-2-picrylhydrazyl (DPPH) radical scavenging activity, reducing power, and reducing total phenolic contents.

## 2. Results and Discussion

### 2.1. Antioxidant Activity and Total Phenolic Contents of C.yabunikkei Extracts

The antioxidant potential of various extracts of the *C.yabunikkei* leaf was determined by measuring 2,2-diphenyl-1-picrylhydrazyl (DPPH) scavenging activity, reducing power assay and reducing total phenolics. The DPPH scavenging assay is a fast and easy method for evaluating the free radical scavenging capacity of given samples. The antioxidant effects of extracts are generally related to the phenolic contents, and phenolic-rich sources of phytochemicals with antioxidant activity have curative benefits against conditions such as inflammation, oxidative stress, and other metabolic diseases [1]. The antioxidative properties of the test plant extracts were closely correlated with the composition of active compounds such as phenolics.

**Figure 1:**
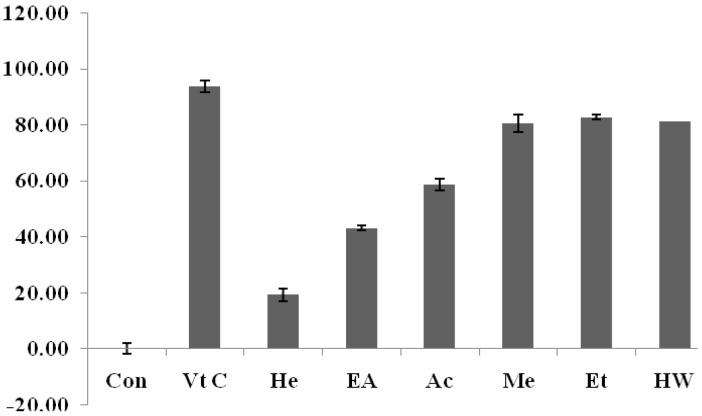
DPPH scavenging activity of solvent extracts of *C.yabunikkei* leaf (extract cont: 0.5 mg/mL, Ascorbic acid: 20 μg/mL). The asterisk represents a value significantly different from the other groups (*p* < 0.05). Values were the mean ± standard deviation (*n* = 3) Vt C; ascorbic acid, He; hexane ex, EA; ethylacetate ez, Ac; acetone ex, Me; methanol ex, Et; ethanol ex, HW; hot water ex.

Therefore, we compared the phenolic contents (mg/g as gallic acid) of various *C.yabunikkei* leaf extracts. The DPPH radicalscavenging activity is shown in Figure 1. The hot water, methanol, and ethanol extracts showed the highest DPPH radical scavenging activity (81.4 ± 0.8%, 80.6 ± 2.1%, 82.89 ± 3.1%) at the concentration of 0.5 mg/mL. Ascorbic acid (Vit C, 20 μg/mL) was used as positive control.

The reducing power assay is useful for evaluating the antioxidant activity. In the present study, the reductive capability of extract samples was tested by measuring the reduction of Fe^3+^. The hot water extract exhibited the highest activity compared to the other extracts (Table 1). The reductive activity expressed as vitamin C equivalents was 3.27 ± 0.052 μg/100 μg as the extract.

The total phenolic content was determined by Folin–Ciocalteu method [1], and the data was reported as gallic acid equivalents by referencing the standard curve (*r*^2^ > 0.999), as shown in Table 1. The phenolic content of the hot water extract was higher than that of the other extracts (16.39 ± 0.28 mg/g as gallic acid equivalents). Taken together, the results indicate that the DPPH radical scavenging activity, power reduction, and phenolic contents reduction were significantly higher in the hot water extract as compared to other extracts.

### 2.2. Xanthine Oxidase Inhibitory Activity of C.yabunikkei Leaf Extracts

**Figure 2:**
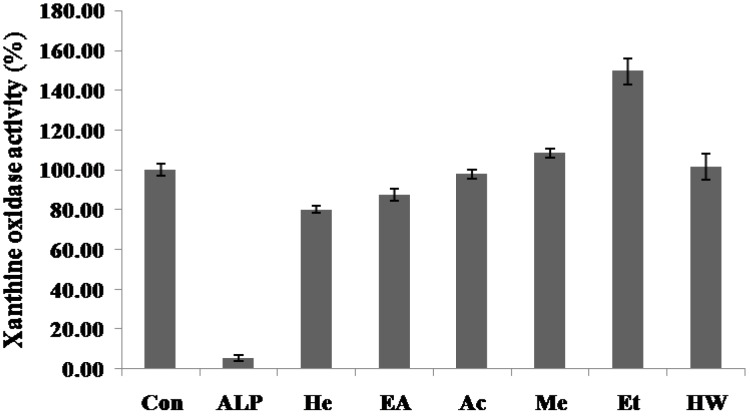
Xanthine oxidase inhibitory activities in various solvent extracts of *C.yabunikkei* leaves (1 mg/mL) and allopurinol (ALP, 50 μg/mL). ALP; allopurinol, He; hexane, EA; ethylacetate, Ac; acetone, Me; methanol, Et; ethanol, HW; hot water extract. Values were the mean ± standard deviation (*n* = 3).

The effect of various solvent extracts on the xanthine oxidase inhibitory activity of the *C.yabunikkei* leaf is shown in Figure 2. Allopurinol (ALP, positive control) at a concentration of 50 μg/mL significantly inhibited xanthine oxidase activity (94.54%). The xanthine oxidase inhibitory activity of the hexane extract was significantly higher than other extracts at the concentration of 1 mg/mL (19.93%). Previously, we reported four different botanical extracts as potential xanthine oxidase inhibitors [2]. Yoon et al. [3,4] reported that the optimized extracts of *Corylopsis coreana* and *Camellia japonica* inhibited xanthine oxidase activity by approximately 50% at a concentration of 2 mg/mL. Additionally, Yoon et al. [2] demonstrated that Quercus acuta extract showed approximately 50% xanthine oxidase inhibitory activity at a concentration of 1 mg/mL. *Cudrania tricuspidata* extract inhibited xanthine oxidase by approximately 75% at a concentration of 2 mg/mL [5]. Previously, we have screened four kinds of xanthine oxidase inhibiting sources in five hundred Korean plant extracts (data not shown). The activities of hexane extract were similar to the extracts of *Corylopsis coreana* and *Camellia japonica*, but about 2.5 times lower than Quercus acuta extract and about 1.8 times lower than *Cudrania tricuspidata* extract. The plant extracts with XO inhibitory activity at 1 and 2 mg/mL demonstrated consistent effects in the in vivo animal disease model. Thus, it is plausible that the hexane extract of *C.yabunikkei* leaves could be developed as a candidate anti-hyperuricemic agent.

### 2.3. Elastase Inhibitory Activity of C.yabunikkei Leaf Extracts

**Figure 3:**
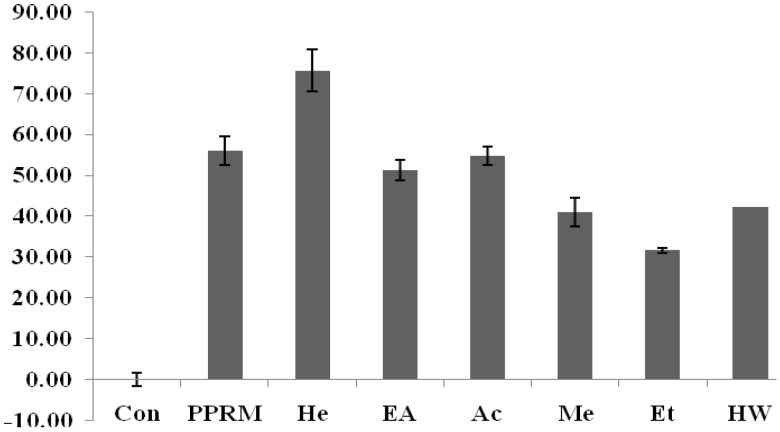
Elastase inhibitory activities in various solvent extracts of *C.yabunikkei* leaves (0.5 mg/mL) and Phospharamidon (PPRM, 0.5 mg/mL), He; hexane, EA; ethylacetate, Ac; acetone, Me; methanol, Et; ethanol, HW; hot water extract. Values were the mean ± standard deviation (*n* = 3).

The effect of various solvent extracts on the elastase inhibitory activity of *C.yabunikkei* leaf is shown in Figure 3. Phosphoramidon (PPRM, positive control) at a concentration of 0.5 mg/mL significantly inhibited elastase activity (56.12 ± 1.53%). The elastase inhibitory activity of the hexane extract was significantly higher than other extracts and positive control at the concentration of 0.5 mg/mL (75.65 ± 3.5%).

### 2.4. Identification of Some Active Constituents

In the present study, several candidates of extracts of *C.yabunikkei* were analyzed and it was confirmed that several compounds’ were present as common constituents inthe *C.yabunikkei* leaves. This finding is of significance in the use of the plant for industrial purposes.

None of the studies reported so far state the diverse activities and constituents of extracts of *C.yabunikkei* leaves. Thus, to the best of our knowledge, our present study is the first to report the optimization of the extraction process of pharmaceutically active indicators from *C.yabunikkei* leaves and the comparison of the antioxidant, xanthine oxidase inhibitory and elastase activities of various extracts.

**Figure 4:**
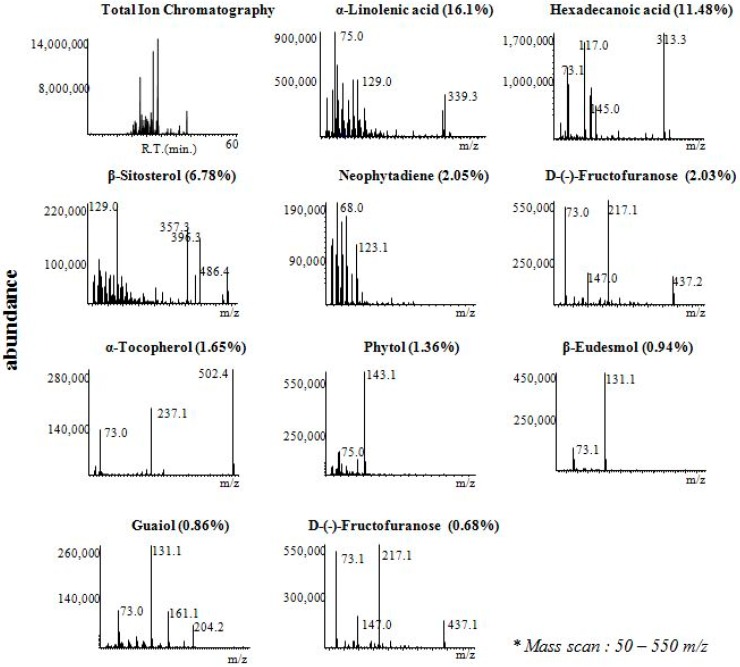
Representative GC-MS chromatogram of extracts of *C.yabunikkei* leaf.

GC-MS analyses were performed to identify the active constituents from *C.yabunikkei* leaf with antioxidant, xanthine oxidase inhibitory and elastase inhibitory activities. Typical GC-MS chromatograms of phytochemical contents and their retention times are shown in Table 2. Eight compounds [i.e., α-Linolenic acid (16.1%), Hexadecanoic acid (11.48%), Neophytadiene (2.05%), d-(−)-Fructofuranose (2.03%), α-Tocopherol (1.65%), Phytol (1.36%), β-Eudesmol (0.94%), Guaiol (0.86%), and d-(−)-Fructofuranose (0.68%)] were mainly identified by GC-MS analysis (Figure 4). As shown in Table 2, several components associated with antioxidant efficacy and xanthine oxidase inhibition were referenced. α-Linolenic acid and α-Tocopherol were typical substances with both antioxidant effect and xanthine oxidase inhibition. In Table 2, it is considered that the activity of hexane extract is due to the synergistic effect of various substances since a number of active substances are involved in the process of antioxidant and xanthine oxidase inhibition.

## 3. Experimental Section

### 3.1. Plant Material and Extract Preparation

*C.yabunikkei* leaves were supplied by Wando Arboretum (Wando, Korea). A voucher specimen (MNUCSS-CY-01) was deposited at the Mokpo National University (Muan, Korea). Air-dried and powdered *C.yabunikkei* leaves (20 g) were subjected to extraction twice with hexane, ethyl acetate, acetone, ethanol, and methanol (100 mL) at room temperature for 48 h or subjected to extraction with hot water (100 °C) for 4 h. The resultant solution was evaporated, dried, and stored at −20 °C for further experiments.

### 3.2. DPPH Free Radical Assay

Antioxidant activity of the sample was determined following a 2,2-diphenyl-1-picrylhydrazyl (DPPH) radical scavenging assay. Briefly, sample solution (1 mL) containing 1 to 20 mg of sample was added to 0.4 mM DPPH sample solution (1 mL) and mixed. The mixture was allowed to react at room temperature in the dark for 10 min. Absorbance value at 517 nm was measured using a microplate reader (Perkin Elmer, Waltham, MA, USA). The DPPH free radical scavenging activities of samples were compared in terms of their IC_50_ (μg/mL) values [5].

### 3.3. Reducing Power

The reducing power of the sample was determined following a modified reducing power assay method. The sample (0.1 mL) was added to 0.2 M sodium phosphate buffer (0.5 mL) and 1% potassium ferricyanide (0.5 mL) followed by incubation at 50 °C for 20 min. Subsequently, 10% trichloroacetic acid solution (0.5 mL) was added to the reaction mixture followed by centrifugation at 12,000 rpm for 10 min. The supernatant was mixed with distilled water (0.5 mL) and 0.1% iron (III) chloride solution (0.1 mL). The absorbance value of the resulting solution was measured at 700 nm. Reducing powers of samples were expressed as vitamin C equivalents [5].

### 3.4. Determination of Total Phenolic Content

The total phenolic content was determined using Folin-Ciocalteu assay [5]. Sample (1 mL) containing 5 mg of sample or standard was mixed with 1 mL of 2% sodium carbonate solution and 1 mL of 10% Folin-Ciocalteu’s phenol reagent. After incubating the mixture at room temperature for 10 min, the absorbance was measured at 750 nm using a microplate reader (Perkin Elmer, Waltham, MA, USA) and compared with the calibration curve of gallic acid. Results were expressed as milligrams of gallic acid equivalents per gram of sample [5].

### 3.5. Determination of Xanthine Oxidase Inhibitory Activity

Xanthine oxidase inhibitory activity was measured by monitoring uric acid formation in the xanthine oxidase system as described previously [5]. The assay system consisted of 0.6 mL phosphate buffer (100 mM; pH 7.4), 0.1 mL sample, 0.1 mL xanthine oxidase (0.2 U/mL), and 0.2 mL xanthine (1 mM; dissolved in 0.1 N NaOH). The reaction was initiated by adding the enzyme with or without inhibitors. A 0.2 mL aliquot of 1 N HCl was used to stop the enzymatic reaction. Allopurinol was used as a positive control. The absorbance of the reaction mixture was measured at 290 nm using a microplate reader (Perkin Elmer, Waltham, MA, USA).

### 3.6. Determination of Elastase Inhibitory Activity

The assay was modified and performed according to the method of Chiocchio et al. [16]. Briefly, 10 μL elastase from porcine pancreas (10 μg/mL) was mixed with 90 of 0.2M Tris-HCl, 100 uL of STANA (2.5 mM, *N*-Succinyl-Ala-Ala-Ala-*p*-nitroanilide), and 50 μL of sample at 37 °C for 30 min. After completion of the reaction, the supernatant was centrifuged at 15,000 rpm for 10 min. The absorbance of the reaction mixture was measured at 405 nm using a microplate reader (Perkin Elmer, Waltham, MA, USA). Phosphoramidon was used as a positive control.

### 3.7. Chemical Profiling by GC-MS Analysis

The molecular mass fragmental scanning of active constituents from *C.yabunikkei* leaf using GC-MS was performed based on a previously reported method with moderate modifications [14]. Briefly, Agilent 7890 gas chromatography (GC) and Agilent 5975 quadrupole mass spectrometry (MS) system (Agilent Technologies, Palo Alto, CA, USA) was utilized to analyze molecular mass fragments (50–550 amu) of *C.yabunikkei* leaf. The mass fragments were ionized under electron ionization (EI) conditions after an Agilent HP-5MS fused silica capillary column (30 mm *l.* × 0.25 mm *i.d.*, 0.25-μm film thickness). GC oven was thermally programmed as isothermal at 65 °C for 10 min and 10 min^−1^ to 300 with helium (He) as a carrier gas. All the scanned mass spectra were compared with the data system library (NIST 2017).

## 4. Conclusions

The present study reveals that hot water extract of *C.yabunikkei* leaf possesses antioxidant activity and hexane extract possesses xanthine oxidase and elastase inhibitory activities. In addition, it is hypothesized that the photochemicals present in the *C.yabunikkei* leaf might be responsible for the biological activities. The results of this study provide an excellent foundation for the future development of *C.yabunikkei* leaf-based dietary or medicinal preparations.

## Figures and Tables

**Table 1:** Reducing power and total phenolic contents of *C.yabunikkei* leaf extracts.

Extract	Reducing Power (Ascorbic Acid eq. μg/100 μg Extract)	Total Phenolic Content (Gallic Acid eq. mg/g)
He	0.25 ± 0.002	0.06 ± 0.00
EA	0.55 ± 0.003	0.98 ± 0.00
Ace	1.28 ± 0.018	6.18 ± 0.19
Me	2.73 ± 0.068	9.24 ± 0.17
Et	2.05 ± 0.034	13.89 ± 0.45
HW	3.27 ± 0.052	16.39 ± 0.28

**Table 2:** Identified substances from the hexane extracts of *C.yabunikkei.*

RT (min)	Hit Name	Quality	M.W.	Composition (%)	Antioxidant Activity [reference]	Xanthine Oxidase Inhibition [reference]
19.681	Methyleugenol	95	178	0.34%		
22.885	Dodecanoic acid	99	272	0.27%		
23.251	Guaiol	91	294	0.86%	[6]	
24.001	β-Eudesmol	91	294	0.94%	[7]	
24.51	d-(−)-Fructofuranose (isomer 2)	91	540	2.03%	[8]	
24.59	d-(−)-Fructofuranose (isomer 1)	93	540	0.68%	[8]	
24.653	d-(−)-Fructopyranose (isomer 1)	93	540	0.65%		
24.956	Neophytadiene	97	278	2.05%	[9]	
25.048	Myristic acid	97	300	0.48%		
25.443	α-d-Mannopyranose	94	540	1.21%		
26.272	β-d-Glucopyranose	95	540	2.59%		
27.039	Hexadecanoic acid	99	328	11.48%	[10]	
27.811	Oleyl alcohol	99	340	0.22%		
27.937	Heptadecanoic acid	96	342	0.49%		
28.109	9(*E*),11(*E*)-Conjugated linoleic acid	99	308	0.59%		
28.189	Phytol	96	368	1.36%	[11]	
28.63	α-Linolenic acid	99	350	16.10%	[12]	[13]
28.819	Octadecanoic acid	99	356	1.07%	[14]	
31.605	1-Monopalmitin	93	474	0.26%		
33.03	Glycerol monostearate	95	502	0.70%		
35.982	α-Tocopherol	99	502	1.65%	[15]	[15]
37.298	Campesterol	99	472	0.67%		
38.449	β-Sitosterol	99	486	6.78%		
